# Synergistic effects of fibrolytic enzymes and lactic acid bacteria on cassava leaf silage quality and microbial community

**DOI:** 10.1186/s40643-026-01151-z

**Published:** 2026-10-24

**Authors:** Md Delowar Hossain, Qiongxian Yan, Suntorn Wittayakun, Viengsakoun Napasirth, Pramote Paengkoum, Zhiliang Tan

**Affiliations:** 1 CAS Key Laboratory of Agro-Ecological Processes in Subtropical Region, National Engineering Laboratory for Pollution Control and Waste Utilization in Livestock and Poultry Production, Hunan Provincial Key Laboratory of Animal Nutrition & Physiology and Metabolism, Institute of Subtropical Agriculture, Chinese Academy of Sciences, Changsha, 410125 People’s Republic Of China; 2 University of Chinese Academy of Sciences, Beijing, 100049 People’s Republic Of China; 3 Department of Animal Science and Nutrition, Gazipur Agricultural University, Gazipur, 1706 Bangladesh; 4 Animal Science and Fishery, Rajamangala University of Technology Lanna, Lampang, 52000 Thailand; 5 Department of Livestock and Fisheries, Faculty of Agriculture, National University of Laos, 856 Vientiane Capital, Laos; 6 School of Animal Technology and Innovation, Institute of Agricultural Technology, Suranaree University of Technology, Nakhon Ratchasima, 30000 Thailand

**Keywords:** Cellulase, Glucanase, Cassava leaf silage, Nutrient composition, Bacterial community

## Abstract

Cassava leaves are a valuable protein-rich feed resource, but their high fiber content and poor fermentation characteristics can reduce silage quality. To address these challenges, this study evaluated the synergistic effects of fiber-degrading enzymes and lactic acid bacteria (LAB) on the fermentation profiles, nutritional quality, and microbial community of cassava leaf silage. Silage was prepared using five treatments: CK (2% glucose), T1 (*Lactobacillus plantarum* + 2% glucose), T2 (glucanase + *L. plantarum* + 2% glucose), T3 (cellulase + *L. plantarum* + 2% glucose), and T4 (glucanase + cellulase + *L. plantarum* + 2% glucose), and ensiled for 0, 21, and 42 days. The findings demonstrated that additive treatments (T1-T4) rapidly decreased silage pH and increased lactic acid compared to the CK (*P* < 0.001). By day 42, the enzyme-inoculated groups (T2-T4) exhibited elevated crude protein alongside reduced fiber fractions compared to both CK and T1 (*P* < 0.001). Regarding the microbial community, Shannon and Simpson indices were lower in treated groups than in the CK group (*P* < 0.05). Beta diversity analysis revealed that the microbial community structure of the CK differed clearly from that of the additive-treated silages. At the phylum level, Bacillota dominated all other groups on day 42, with a relative abundance exceeding 92%. At the species level, *Lactobacillus plantarum* dominated enzyme-treated silages (relative abundance > 93%, *P* = 0.036), compared to only 13.1% in CK, which contained higher proportions of *Pediococcus* and *Weissella* species. Collectively, the synergistic application of fibrolytic enzymes and LAB improves cassava leaf silage quality by accelerating acidification, reducing fiber fractions, and establishing a highly favorable *L. plantarum-*dominated microbial community.

**Supplementary Information:**

The online version contains supplementary material available at https://doi.org/10.1186/s40643-026-01151-z.

## Introduction

The escalating global demand for sustainable and high-quality animal feed, particularly in tropical and subtropical regions, has intensified the search for alternative, cost-effective, and non-human-competing feed resources. Cassava (*Manihot esculenta* Crantz), a staple crop cultivated extensively for its starchy tubers (Burns et al. 2010), generates substantial quantities of leafy biomass as a by-product (Wu Qiufei et al. 2017). Annually, millions of tons of cassava foliage are either left to decompose in fields or burned, representing a significant loss of potential biomass (Rashid et al. 2025). Cassava foliage contains a considerable amount of crude protein (approximately 16–40%), essential minerals, and vitamins, positioning it as a potentially valuable ruminant feed resource (Hossain et al. 2025). However, their utilization as fresh forage is severely constrained by the presence of cyanogenic glycosides, which can release toxic hydrogen cyanide (HCN) upon ingestion (Ndubuisi and Ano 2018). Furthermore, the high fiber content and rapid post-harvest deterioration due to high moisture content limit their direct inclusion in animal diets.

Ensiling remains one of the most cost-effective biotechnological approaches to reduce HCN and preserve moist biomass while enhancing its nutritional profile (Man Ngo Van & Hans 2001). The success of ensiling is fundamentally dependent on sufficient populations of desirable microorganisms and adequate fermentable substrates. However, cassava leaves generally contain low levels of epiphytic lactic acid bacteria and limited water-soluble carbohydrates (WSC), which may result in poor fermentation and undesirable microbial activity during ensiling (Li et al. 2019). To overcome these limitations, microbial inoculants and fibrolytic enzymes are commonly used as silage additives. Fibrolytic enzymes, including cellulase, xylanase, and β-glucanase, hydrolyze structural polysaccharides into fermentable sugars, thereby increasing substrate availability for fermentation while simultaneously improving fiber digestibility (Dagaew et al. 2025; Liu et al. 2023). Concurrently, LAB inoculants accelerate pH decline via homo-or hetero-fermentative pathways, thereby preserving nutrients and inhibiting undesirable microbial activity (Bai et al. 2024; Vuma et al. 2026). Napasirth et al. (2015) reported that inoculation with LAB enhanced the fermentation of cassava residues, as indicated by lowering pH and elevated lactic acid concentrations compared with untreated controls. Similarly, Li et al. (2019) reported that cassava foliage silage treated with *Lactobacillus plantarum* or *L. rhamnosus* in combination with Acremonium cellulase exhibited superior fermentation characteristics, along with enhanced crude protein preservation and reduced fiber contents.

The combined use of fibrolytic enzymes and LAB has gained increasing interest due to their potential synergistic effects during the ensiling process. Enzymatic hydrolysis provides additional soluble carbohydrates for LAB growth (Wang et al. 2019), while rapid acidification by LAB creates favorable conditions for silage preservation (Kim et al. 2021). A recent meta-analysis by Liu et al. (2026), encompassing 250 studies, confirmed that application of fiber-degrading enzymes in combination with LAB led to the most significant decreases in pH, neutral detergent fiber (NDF), acid detergent fiber (ADF), and hemicellulose content compared with either treatment alone, while simultaneously enhancing lactic acid accumulation and reducing microbial diversity indices. In high-moisture alfalfa silage, Chen et al. (2019) reported that the combination of LAB inoculants (*L. plantarum* and *Pediococcus pentosaceus*) with fiber-degrading enzymes enhanced homo-fermentation efficiency and improved in vitro dry matter degradability compared to individual treatment. Li et al. (2019) reported that the combined use of cellulase and *Lactobacillus plantarum* produced better fermentation characteristics and ruminal degradability in cassava foliage silage compared with individual treatment. Similar synergistic effects were also observed in sorghum straw silage and mixed forage silages, where combined treatments resulted in lower pH, higher lactic acid content, and improved nutrient preservation (He et al. 2025; Wang et al. 2022).

Despite accumulating evidence supporting the benefits of combined enzyme-LAB application across various forages, the specific synergistic effects of a dual fibrolytic enzyme comprising cellulase and glucanase, together with LAB inoculation, on cassava leaf silage quality and microbial community remain inadequately characterized. Although several studies have evaluated the individual or combined effects of LAB and fibrolytic enzymes on traditional forages (e.g., corn or alfalfa), where fermentation dynamics differ fundamentally (Chen et al. 2019; Li et al. 2023). Most existing studies on cassava foliage have focused on single enzyme or LAB treatments, with limited investigation of the mechanistic interactions between these additives and the indigenous epiphytic microbial community (Bakare et al. 2023; Li et al. 2021; Liu et al. 2022a). Therefore, this study was designed to test the hypothesis that the combined use of cellulase, glucanase, and selected LAB strains produced beneficial synergistic effects on fermentation performance, nutritional quality, and bacterial community development in cassava leaf silage. These results may support the improved preservation and utilization of cassava leaves as a high-quality feed resource for ruminants in tropical and subtropical areas.

## Materials and methods

### Experimental materials

The test materials used for cassava leaf silage preparation included fresh cassava leaves (with stems and petioles) as the primary substrate. Cassava plants were grown on the experimental land of the Institute of Subtropical Agriculture located in Taoyuan County, Hunan, China (111°27′E, 28°55′N). Glucose (analytical grade; Sinopharm Chemical Reagent Co. Ltd., China) was added as a fermentable carbohydrate source. A commercial strain of *Lactobacillus plantarum* (more than 1 × 10^10^ CFU/g; Shandong Bioengineering Co. Ltd., China) was used as the microbial inoculant to promote lactic acid fermentation. Cellulase (16,000 U/g; Wuhan Sunhy Biology Co. Ltd., China) and glucanase (50,000 U/g; Wuhan Sunhy Biology Co. Ltd., China) were applied as fibrolytic enzymes to enhance the degradation of structural carbohydrates. All materials were thoroughly mixed according to the experimental treatments before ensiling under anaerobic conditions.

### Experimental design

A completely randomized design (CRD) was used to assess the effects of microbial inoculation and enzymatic supplementation on cassava leaf silage. Five treatments were established: CK (2% glucose), T1 (*Lactobacillus plantarum* at 3 × 10^8^ CFU/g + 2% glucose), T2 (glucanase at 833 U/g + *L. plantarum* at 3 × 10^8^ CFU/g + 2% glucose), T3 (cellulase at 533 U/g + *L. plantarum* at 3 × 10^8^ CFU/g + 2% glucose), and T4 (glucanase at 833 U/g + cellulase at 533 U/g + *L. plantarum* at 3 × 10^8^ CFU/g + 2% glucose). Each treatment was replicated four times to minimize variability due to environmental and handling differences.

### Silage preparation

Cassava leaves, including young stems and petioles, were harvested when the plant reached approximately 1–1.5 m in height. The leaves and stems were cut into small pieces of about 2–3 cm with a grass chopper and then allowed to wilt for 4 h at ambient temperature until the moisture content reached around 60% of DM. After wilting, the silage was prepared in accordance with the experimental design. Cellulase and glucanase enzymes were added in 10 g and 5 g per bag, respectively. All the treated materials were then thoroughly mixed and tightly packed into plastic bags (30 cm × 20 cm), evacuated, and sealed with a vacuum machine (Anshengke DZQ-390, China). A total of 60 bags (five treatments × three ensiling days × four replications) were individually prepared, and each bag contained 300 g of silage. After sealing, the bags were kept at room temperature under cool and dry storage conditions to allow proper fermentation. The silage was allowed to ferment for 0, 21, and 42 days to assess fermentation characteristics and nutritive value.

### Determination of fermentation indices

The fermentation quality of silage was evaluated by determining key fermentation indices, particularly pH and organic acid concentrations. Silage pH was determined by diluting a 10 g fresh sample in 90 mL distilled water, letting it stand for 1 h, and measuring with a calibrated pH meter (PH 50, Watenv Technology Co. Ltd., Shanghai, China). Acetic and propionic acids were measured by gas chromatography (Agilent 7890 A) fitted with a DB-FFAP column (30 m × 0.25 mm × 0.25 μm); injector temperature was 250 °C, and the oven ramped from 60 °C (2 min hold) to 220 °C at 20 °C/min (1.5 min hold), with nitrogen carrier gas at 0.8 mL/min and a 1.0 µL injection. Lactic acid was quantified by HPLC (Agilent 1260) using a Zorbax SB-C_18_ column (4.6 × 150 mm, 5 μm) at 25 °C, with a phosphate buffer/acetonitrile mobile phase (98:2, isocratic), 1.0 mL/min flow rate, 20 µL injection, 10 min runtime, and detection at 210 nm. Organic acids were identified and quantified against external standards using retention times and peak areas.

### Determination of nutrient composition and fiber components

The nutrient composition and fiber components of cassava leaves silage were determined using standard analytical procedures. After thorough mixing, fermented cassava leaf samples were oven-dried (65 °C, 72 h), then ground and passed through a 0.3 mm sieve for compositional analysis. Dry matter (DM) was determined by drying at 65 °C to constant weight, with a subsequent 105 °C step to capture total moisture. Crude protein (CP) was estimated from Kjeldahl nitrogen (N × 6.25), and ether extract (EE) was determined via Soxhlet extraction with petroleum ether (AOAC 2000). NDF and ADF were assayed following Van Soest et al. (1991) on a Fibertherm fiber analyzer (Gerhardt Analytical Systems, Germany). Acid detergent lignin (ADL) was obtained by treating ADF residue with 72% sulfuric acid (Goering and Van Soest 1970); hemicellulose (NDF − ADF) and cellulose (ADF − ADL) were derived from these values. WSC were assayed by anthrone-sulfuric acid colorimetry (Leyva et al. 2008), at 625 nm (TECAN Infinite M200 PRO microplate reader) against a glucose standard curve. Ash was quantified by muffle furnace incineration at 550 °C for 3–4 h to constant weight. Relative feed value (RFV) was calculated from digestible dry matter (DDM) and dry matter intake (DMI), where DDM = 88.9 − (0.779 × ADF) and DMI = 120/NDF. RFV was calculated as (DDM × DMI)/1.29.

### Microbial diversity analysis

Genomic DNA was extracted from the 42-day silage samples with the E.Z.N.A.^®^ Soil DNA Kit (Omega Bio-tek, USA), and its quality and concentration were verified by NanoDrop 2000 spectrophotometer and 1% agarose gel electrophoresis. The bacterial 16 S rRNA V3–V4 region was amplified with primers 341 F (5′-CCTAYGGGRBGCASCAG-3′) and 806R (5′- GGACTACNNGGGTATCTAAT-3′). PCR products were quantified with the QuantiFluor™-ST system after preliminary electrophoresis, then pooled proportionally per sequencing requirements. Libraries were built with the NEXTFLEX Rapid DNA-Seq Kit and sequenced as paired-end reads on BGI’s DNBSEQ- G99 platform . Raw reads were processed in QIIME2, with amplicon sequence variants (ASVs) generated and denoised via DADA2 (Callahan et al. 2016). Taxonomic classification of the resulting ASVs was performed utilizing the ‘uclust’ algorithm (Edgar 2010) against the Silva SSU138.1 database (Quast et al. 2013), applying a 0.8 confidence cutoff (Kim and Chun 2014) from phylum to species level. For alpha-diversity, rarefaction analysis was performed using Mothur v1.21.1 (Schloss et al. 2009), and beta-diversity was examined via UniFrac distance matrix visualized by non-metric multidimensional scaling (NMDS) using the vegan package in R-forge.

### Statistical analysis

Data were analyzed in SPSS 26.0 using a general linear model (GLM) procedure. A two-way ANOVA tested treatment, ensiling duration, and their interaction on fermentation and nutritional parameters. Duncan’s multiple range test was used for mean comparisons, with significance set at *P* < 0.05. Differences in bacterial abundance were evaluated using the non-parametric Kruskal-Wallis test. Spearman’s correlation analysis was used to examine associations between bacterial species, fermentation characteristics, and nutrient composition, and the results were visualized using a correlation heatmap. Results are presented as means together with their standard errors (SEM).

## Results

### Fermentation quality of cassava leaf silage

The pH values showed a significant decline over the ensiling period (*P* < 0.001), with the lowest pH observed on Day 42 across all treatments (Table 1). The control (CK) maintained the higher pH values throughout fermentation, whereas enzyme- and LAB-treated silage showed markedly lower pH values. Among the treatments, T2 exhibited the lowest pH (3.88) at Day 21 and (3.78) at Day 42, demonstrating improved acidification and fermentation efficiency compared with the CK. In contrast, lactic acid concentration increased throughout the ensiling period (*P* < 0.001). At Day 42, the treated silages produced significantly more lactic acid (5.92–6.03%) than the CK (4.56%), with T1 showing the highest value (6.03%). Acetic acid concentration was influenced by both treatments and ensiling time (*P* < 0.001). The control treatment showed relatively stable acetic acid content (0.31–0.35%) throughout the fermentation process, whereas treatments T1-T4 initially exhibited higher levels (0.43–0.49%) that declined sharply by Day 21 (*P* < 0.001). Propionic acid concentration remained comparatively low in all treatments and increased slightly over time, from approximately 0.010 to 0.013% at Day 0 to 0.020–0.024% at Day 42. Treatment (*P* = 0.015) and ensiling duration (*P* < 0.001) significantly affected propionic acid concentration. Butyric and iso-butyric acids were not detected in any treatment.

**Table 1:** Effects of the additives on the fermentation parameters of cassava leaf silage

Item	Treatment	Ensiling days			SEM	*P*-Value		
		0	21	42		T	D	T×D
pH	CK	5.56 A	4.53 Ba	4.41 Ca	0.10	< 0.001	< 0.001	< 0.001
	T1	5.54 A	4.05 Bb	3.94 Cb				
	T2	5.57 A	3.88 Bc	3.78 Cd				
	T3	5.59 A	3.89 Bc	3.80 Ccd				
	T4	5.58 A	3.90 Bc	3.81 Cc				
Lactic acid (% DM)	CK	4.38 b	4.46 b	4.56 b	0.09	< 0.001	< 0.001	< 0.001
	T1	4.74 Ba	5.79 Aa	6.03 Aa				
	T2	4.67 Ca	5.56 Ba	5.95 Aa				
	T3	4.54 Bab	5.69 Aa	5.94 Aa				
	T4	4.58 Cab	5.71 Ba	5.92 Aa				
Acetic acid (% DM)	CK	0.31 Bc	0.35 Aa	0.34 ABa	0.01	< 0.001	< 0.001	< 0.001
	T1	0.44 Ab	0.24 Bd	0.26 Be				
	T2	0.43 Ab	0.25 Bcd	0.27 Bd				
	T3	0.47 Aab	0.27 Bbc	0.29 Bc				
	T4	0.49 Aa	0.28 Cb	0.32 Bb				
Propionic acid (% DM)	CK	0.013 C	0.019 Ba	0.024 Aa	0.001	0.015	< 0.001	0.544
	T1	0.013 C	0.019 Bab	0.022 Aab				
	T2	0.012 B	0.018 Aab	0.020 Ab				
	T3	0.010 C	0.017 Bab	0.022 Aab				
	T4	0.011 C	0.016 Bb	0.022 Aab				

DM: dry matter; SEM: standard error of the means; CK: 2% glucose; T1: *Lactobacillus plantarum* at 3 × 10^8^ CFU/g + 2% glucose; T2: Glucanase at 833 U/g + *L. plantarum* at 3 × 10^8^ CFU/g + 2% glucose; T3: Cellulase at 533 U/g + *L. plantarum* at 3 × 10^8^ CFU/g + 2% glucose; T4: Glucanase at 833 U/g + cellulase at 533 U/g + *L. plantarum* at 3 × 10^8^ CFU/g + 2% glucose; T: effect of enzyme additives; D: effect of ensiling days; D×T: interaction of ensiling days and enzyme additives. Means with different uppercase letters (A-C) within a row and lowercase letters (a-e) within a column differ significantly (*P* < 0.05)

### Chemical composition and fiber components of cassava leaf silage

The nutrient composition of cassava leaf silage across different treatments and ensiling durations is summarized in Table 2. DM content decreased significantly as ensiling time increased across all treatments (*P* < 0.001). At 42 days of ensiling, the enzyme-treated silages (T2-T4) showed lower DM values compared with CK and *L. plantarum-*treated silage (T1). Initially, DM ranged from 37.9 to 39.2%, and by Day 42, it decreased to 31.9–35.1%, with T3 showing the lowest DM. In contrast, CP increased significantly during ensiling, peaking in T4 on the final day (27.7%), while treatments T2-T4 generally had higher CP than CK at Day 42 (*P* < 0.001). Ash content changed significantly (*P* < 0.001) over time and among treatments, particularly in T3 and T4, with the highest value recorded in T4 (12.8%) at Day 42. EE showed a slight but constant increase during fermentation (*P* < 0.001). WSC decreased significantly with prolonged ensiling, reflecting microbial utilization (*P* < 0.001). Among the treatments, T2 retained a relatively higher WSC content (9.84%) after 42 days of ensiling, whereas CK had the lowest (2.40%). RFV was markedly improved by the enzyme treatment and longer ensiling periods (*P* < 0.001). RFV increased from 68.3 in the CK at Day 0 to139 in T4 at Day 42.

**Table 2:** Effects of the additives on the basic nutritional quality of cassava leaf silage

Item	Treatment	Ensiling days			SEM	*P*-Value		
		0	21	42		T	D	T×D
DM (%)	CK	37.9 Ab	37.3 Bb	35.1 Ca	0.30	< 0.001	< 0.001	< 0.001
	T1	38.1 Ab	37.5 Aab	34.4 Ba				
	T2	38.3 Ab	37.3 Bb	32.6 Cbc				
	T3	38.0 Ab	36.9 Bb	31.9 Cc				
	T4	39.2 Aa	37.9 Ba	33.1 Cb				
CP (% DM)	CK	25.4 Bba	25.6 Ba	26.1 Ac	0.16	< 0.001	< 0.001	< 0.001
	T1	24.6 Bb	24.8 Bb	26.6 Abc				
	T2	24.9 Bab	24.4 Bb	27.2 Aab				
	T3	23.8 Bc	24.9 Bb	27.3 Aab				
	T4	25.4 Ba	25.6 Ba	27.7 Aa				
Ash (% DM)	CK	9.88 Bb	9.71 Bc	10.5 Ac	0.16	< 0.001	< 0.001	< 0.001
	T1	8.61 Bd	8.38 Be	9.43 Ae				
	T2	9.06 Cc	9.31 Bd	10.1 Ad				
	T3	10.6 Ca	11.2 Bb	11.9 Ab				
	T4	10.8 Ca	11.7 Ba	12.8 Aa				
EE (% DM)	CK	6.27 Bb	6.43 Ac	6.51 Ad	0.03	< 0.001	< 0.001	0.032
	T1	6.43 Bab	6.54 Bc	6.70 Ac				
	T2	6.50 Ba	6.76 Ab	6.89 Ab				
	T3	6.49 Ba	6.84 Aab	6.86 Ab				
	T4	6.49 Ca	6.90 Ba	7.05 Aa				
WSC (% DM)	CK	11.9 Ac	2.92 Bd	2.40 Bd	0.69	< 0.001	< 0.001	< 0.001
	T1	18.2 Ab	8.96 Bc	7.16 Cc				
	T2	19.8 Aa	12.9 Ba	9.84 Ca				
	T3	18.2 Ab	11.0 Bb	8.08 Cb				
	T4	18.4 Ab	10.6 Bb	7.34 Cc				
RFV	CK	68.3 Ce	70.9 Bd	73.1 Ae	3.33	< 0.001	< 0.001	< 0.001
	T1	73.8 Bd	77.6 Ac	77.9 Ad				
	T2	90.5 Bc	121 Ab	124 Ac				
	T3	99.7 Bb	125 Ab	130 Ab				
	T4	105 Ba	134 Aa	139 Aa				

DM: dry matter; CP: crude protein; EE: ether extract; WSC: water soluble carbohydrates; RFV: relative feed value; SEM: standard error of the means; CK: 2% glucose; T1: *Lactobacillus plantarum* at 3 × 10^8^ CFU/g + 2% glucose; T2: Glucanase at 833 U/g + *L. plantarum* at 3 × 10^8^ CFU/g + 2% glucose; T3: Cellulase at 533 U/g + *L. plantarum* at 3 × 10^8^ CFU/g + 2% glucose; T4: Glucanase at 833 U/g + cellulase at 533 U/g + *L. plantarum* at 3 × 10^8^ CFU/g + 2% glucose; T: effect of enzyme additives; D: effect of ensiling days; D×T: interaction of ensiling days and enzyme additives. Means with different uppercase letters (A-C) within a row and lowercase letters (a-e) within a column differ significantly (*P* < 0.05)

NDF, ADF, cellulose, and hemicellulose contents decreased notably with enzyme addition, especially in T3 and T4, showing strong treatment effects (*P* < 0.001). The reductions became more pronounced with prolonged ensiling. At 42 days, T4 exhibited the lowest NDF (46.8% DM), ADF (24.3% DM), cellulose (14.8% DM), and hemicellulose (22.5% DM) contents compared with the CK (Table 3). Similar trends were observed in T2 and T3, indicating that enzyme supplementation enhanced fiber degradation during fermentation. ADL content decreased slightly during ensiling, although the effects of ensiling days and treatment interaction were not significant (*P* > 0.05). ADL content remained relatively stable, ranging from 9.08 to 11.4%, indicating limited lignin degradation.

**Table 3:** Effects of the additives on the fiber components of cassava leaf silage

Item	Treatment	Ensiling days			SEM	*P*-Value		
		0	21	42		T	D	T×D
NDF (% DM)	CK	77.2 Aa	74.7 Ba	73.4 Ba	1.42	< 0.001	< 0.001	< 0.001
	T1	72.8 b	70.5 b	70.9 b				
	T2	63.8 Ac	51.9 Bc	51.2 Bc				
	T3	58.4 Ad	50.4 Bc	48.8 Bd				
	T4	56.3 Ad	48.0 Bd	46.8 Be				
ADF (% DM)	CK	41.4 a	41.1 a	40.2 a	0.83	< 0.001	< 0.001	< 0.001
	T1	39.9 Aa	38.7 ABb	37.9 Bb				
	T2	34.5 Ab	27.1 Bc	26.1 Bc				
	T3	33.8 Abc	26.7 Bc	26.2 Bc				
	T4	32.1 Ac	25.2 Bd	24.3 Bd				
ADL (% DM)	CK	11.4 a	11.2 a	10.9 a	0.12	< 0.001	0.299	0.849
	T1	10.6 ab	10.9 ab	10.3 ab				
	T2	10.0 bc	10.2 bc	9.51 b				
	T3	9.72 cd	9.76 c	9.81 ab				
	T4	9.08 d	9.53 c	9.49 b				
Cellulose (% DM)	CK	29.9 a	29.9 a	29.3 a	0.77	< 0.001	< 0.001	< 0.001
	T1	29.3 a	27.7 b	27.6 a				
	T2	24.5 Ab	16.9 Bc	16.6 Bb				
	T3	24.1 Ab	16.9 Bc	16.4 Bb				
	T4	23.0 Ab	15.6 Bc	14.8 Bb				
Hemicellulose (% DM)	CK	35.8 Aa	33.5 Ba	33.2 Ba	0.65	< 0.001	0.001	0.447
	T1	32.9 ab	31.8 a	32.9 a				
	T2	29.3 Ab	24.9 Bb	25.1 Bb				
	T3	24.5 c	23.8 b	22.6 c				
	T4	24.2 c	22.9 b	22.5 c				

NDF: neutral detergent fiber; ADF: acid detergent fiber; ADL: acid detergent lignin; SEM: standard error of the means; CK: 2% glucose; T1: *Lactobacillus plantarum* at 3 × 10^8^ CFU/g + 2% glucose; T2: Glucanase at 833 U/g + *L. plantarum* at 3 × 10^8^ CFU/g + 2% glucose; T3: Cellulase at 533 U/g + *L. plantarum* at 3 × 10^8^ CFU/g + 2% glucose; T4: Glucanase at 833 U/g + cellulase at 533 U/g + *L. plantarum* at 3 × 10^8^ CFU/g + 2% glucose; T: effect of enzyme additives; D: effect of ensiling days; D×T: interaction of ensiling days and enzyme additives. Means with different uppercase letters (A-C) within a row and lowercase letters (a-e) within a column differ significantly (*P* < 0.05)

### Dynamic alpha and beta diversity of cassava leaf silage microbiota

**Fig. 1:**
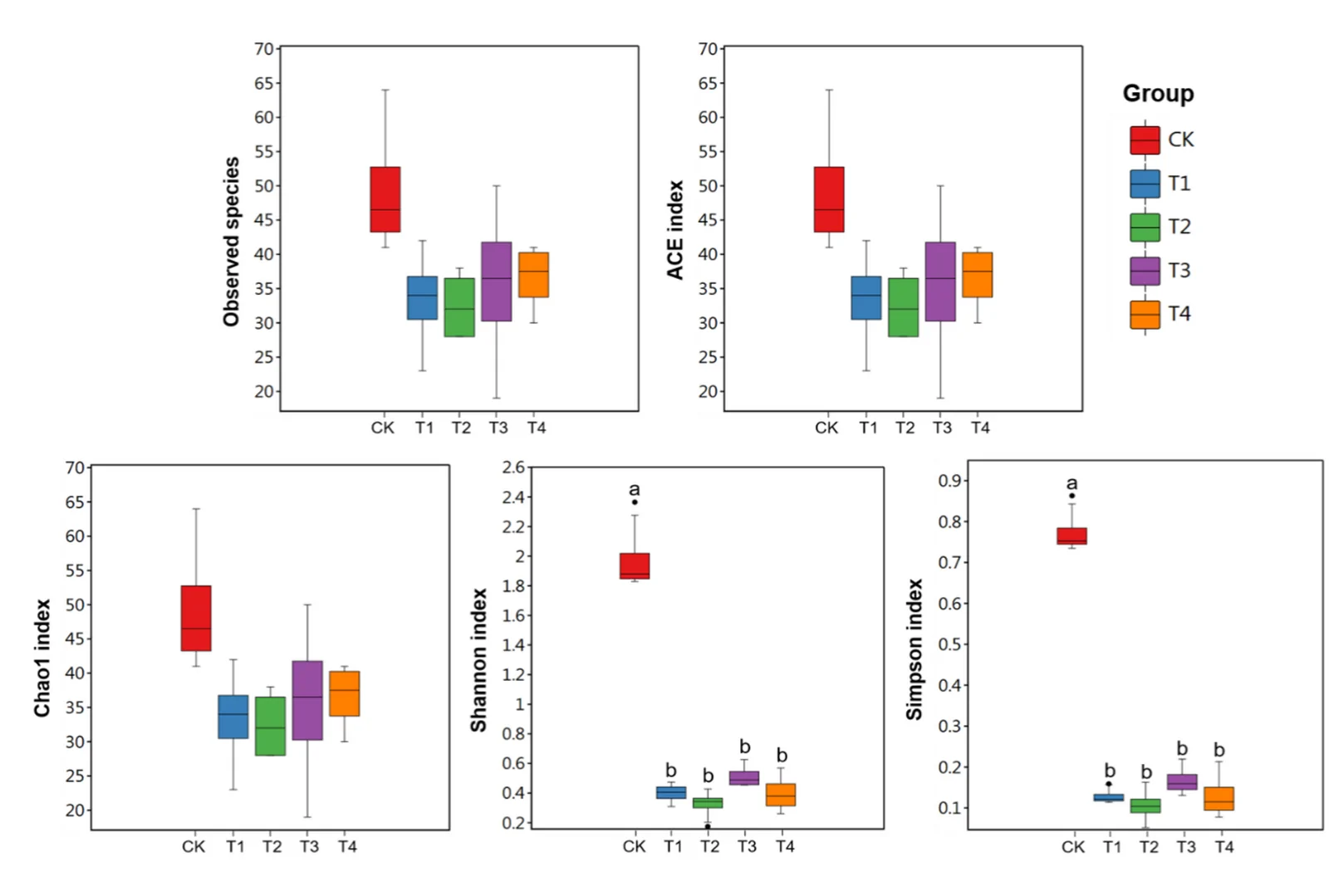
Alpha diversity measures of bacterial community in cassava leaf silage. Each box in the figure represents a group. The horizontal line inside the box represents the median, while the top and bottom lines of the box represent the upper and lower quartiles; black dots outside the box are outliers. The lowercase letters above the boxes indicate the results of inter-group significance tests (*P* < 0.05). CK: 2% glucose; T1: *Lactobacillus plantarum* at 3 × 10^8^ CFU/g + 2% glucose; T2: Glucanase at 833 U/g + *L. plantarum* at 3 × 10^8^ CFU/g + 2% glucose; T3: Cellulase at 533 U/g + *L. plantarum* at 3 × 10^8^ CFU/g + 2% glucose; T4: Glucanase at 833 U/g + cellulase at 533 U/g + *L. plantarum* at 3 × 10^8^ CFU/g + 2% glucose

The alpha diversity indices of the bacterial communities in cassava leaf silage are presented in Fig. 1. Species richness metrics (ACE, Chao1, and observed species) showed non-significant variation between CK and additive-treated groups (T1-T4) (p> 0.05), indicating that the treatments did not substantially affect bacterial richness. In contrast, the Shannon and Simpson indices differed significantly among treatments (*P* < 0.05). The CK treatment showed the highest Shannon and Simpson values, indicating greater bacterial diversity and community evenness. However, the treated silages (T1-T4) exhibited significantly lower values for both indices, with no significant differences among the treated groups.

**Fig. 2:**
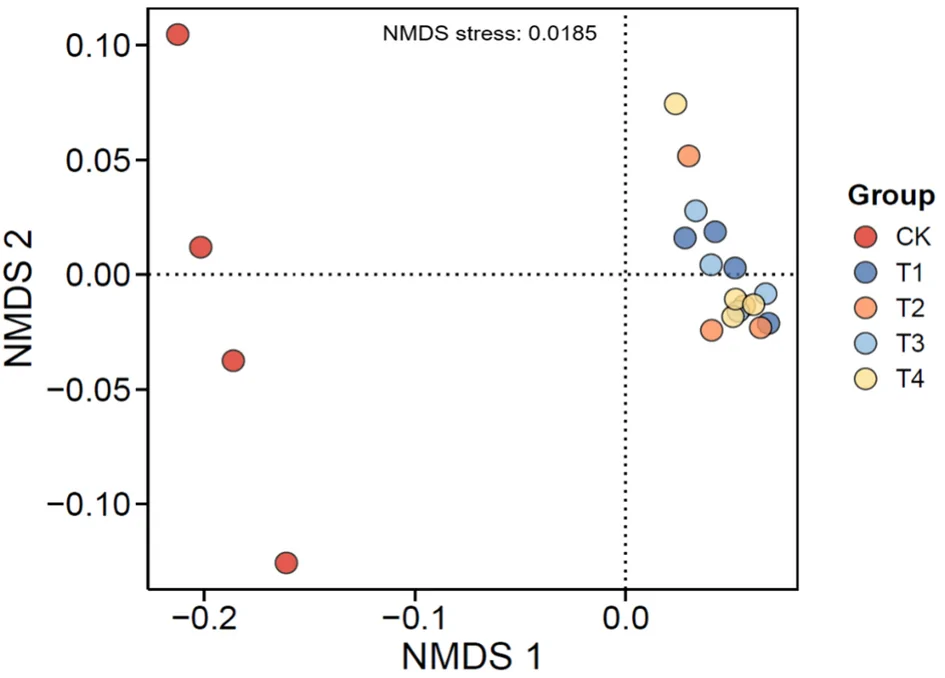
Non-metric multidimensional scaling (NMDS) analysis of cassava leaf silage bacterial community in different groups. Each point in the figure represents a sample, and the distance between points indicates the degree of difference. CK: 2% glucose; T1: *Lactobacillus plantarum* at 3 × 10^8^ CFU/g + 2% glucose; T2: Glucanase at 833 U/g + *L. plantarum* at 3 × 10^8^ CFU/g + 2% glucose; T3: Cellulase at 533 U/g + *L. plantarum* at 3 × 10^8^ CFU/g + 2% glucose; T4: Glucanase at 833 U/g + cellulase at 533 U/g + *L. plantarum* at 3 × 10^8^ CFU/g + 2% glucose

The NMDS analysis using weighted UniFrac distances revealed distinct differences in the bacterial community structure of cassava leaf silage among treatments **(**Fig. 2**)**. The NMDS plot showed a very low stress value (0.0185), indicating that the two-dimensional ordination accurately represented the variation in microbial communities. The CK formed a distinct cluster and was clearly separated from the treated groups along the NMDS1 axis, suggesting a substantial shift in bacterial community composition following additive application. The CK samples clustered on the negative side of NMDS1 and showed greater dispersion among replicates, indicating greater variability in bacterial community structure. In contrast, samples from T1, T2, T3, and T4 were grouped on the positive side of NMDS1 and exhibited relatively compact clustering, indicating more homogeneous microbial communities among these treatments than in the CK. A slight separation among the treated groups was also observed, reflecting treatment-specific effects on microbial community composition.

### Relative abundance of cassava leaf silage bacteria

The phylum-level bacterial composition of cassava leaf silage at 42 days of ensiling was dominated by Bacillota, with relative abundances ranging from 92.2 to 96.1% (Table 4). The treated groups showed a higher abundance of Bacillota than the CK. However, this variation was not statistically significant (*P* > 0.05). Pseudomonadota constituted the second largest proportion, accounting for 3.73–7.35% of the total community, with lower abundance in the treated silages than in CK. Cyanobacteriota, Actinomycetota, and Deinococcota were detected at low abundances (< 0.30%) but were significantly affected by treatment (*P* < 0.05). These phyla were generally less abundant in the treated silages than in the CK. Other phyla, including Verrucomicrobiota, Campylobacterota, Gemmatimonadota, Bacteroidota, and Fusobacteriota, were present in very low proportions and did not vary significantly across treatments (*P* > 0.05).

**Table 4:** Relative abundance of cassava leaf silage bacteria at the phylum level at 42 days of ensiling

Phylum	Treatment					SEM	*P*-value
	CK	T1	T2	T3	T4		
Bacillota	92.2	95.9	95.6	96.1	95.5	0.63	0.299
Pseudomonadota	7.35	3.75	3.74	3.73	4.21	0.60	0.456
Verrucomicrobiota	0.03	0.02	0.51	0.01	0.02	0.10	0.504
Cyanobacteriota	0.26	0.03	0.01	0.04	0.06	0.03	0.026
Campylobacterota	0.04	0.15	0.03	0.03	0.12	0.02	0.152
Actinomycetota	0.08	0.01	0.02	0.02	0.02	0.01	0.031
Deinococcota	0.07	0.01	0.01	0.01	0.01	0.01	0.023
Gemmatimonadota	0.01	ND	0.03	0.03	0.03	0.01	0.527
Bacteroidota	0.01	0.03	0.01	ND	ND	0.00	0.059
Fusobacteriota	ND	0.01	ND	ND	0.01	0.00	0.529

ND: not detected; CK: 2% glucose; T1: *Lactobacillus plantarum* at 3 × 10^8^ CFU/g + 2% glucose; T2: Glucanase at 833 U/g + *L. plantarum* at 3 × 10^8^ CFU/g + 2% glucose; T3: Cellulase at 533 U/g + *L. plantarum* at 3 × 10^8^ CFU/g + 2% glucose; T4: Glucanase at 833 U/g + cellulase at 533 U/g + *L. plantarum* at 3 × 10^8^ CFU/g + 2% glucose; SEM: standard error of the means

The species-level bacterial community of cassava leaf silage after 42 days of ensiling showed significant differences among the treatments (Table 5). *Lactobacillus plantarum* was the predominant species in all treated silages (T1-T4), with relative abundances ranging from 93.0–94.6%, compared with 13.1% in the CK. This rise reached statistical significance (*P* = 0.036), indicating that the additives strongly promoted the growth of *L. plantarum*. In contrast, the abundance of *Pediococcus acidilactici* and *Pediococcus pentosaceus* decreased significantly from 35.8% in CK to 0.03–0.76% and 23.7% in CK to 0.00–0.61%, respectively, in the treated silages. *Weissella cibaria*, *Lactobacillus fermentum*, and *Enterococcus mundtii ATCC 882* also showed significantly lower relative abundances in the treated silage than in CK (*P* < 0.05). Potentially undesirable bacteria such as *Klebsiella pneumoniae* (1.23%) and *Enterococcus faecium* (0.64%) were detected only in the CK and were completely absent in the treated groups (*P* < 0.01). Small proportions of *Bacillus licheniformis* were detected in T3 (0.42%) and T4 (0.73%), and these proportions differed significantly among treatments (*P* = 0.003). In contrast, *Weissella confusa* remained at very low abundance in CK and T2 and exhibited no significant variation (*P* > 0.05).

**Table 5:** Relative abundance of cassava leaf silage bacteria at the species level (top 10) at 42 days of ensiling

Species	Treatment					SEM	*P*-value
	CK	T1	T2	T3	T4		
*Lactobacillus plantarum*	13.1	93.5	94.6	93.0	93.3	7.41	0.036
*Pediococcus acidilactici*	35.8	0.36	0.21	0.76	0.03	3.43	0.007
*Pediococcus pentosaceus*	23.7	0.40	0.11	0.61	ND	2.19	0.002
*Weissella cibaria*	9.40	0.25	0.14	0.29	ND	1.21	0.003
*Lactobacillus fermentum*	4.07	0.08	0.02	0.14	0.01	0.39	0.002
*Enterococcus mundtii ATCC 882*	3.49	0.07	0.03	0.09	ND	0.33	0.004
*Klebsiella pneumoniae*	1.23	ND	ND	ND	ND	0.12	0.001
*Bacillus licheniformis*	0.01	ND	0.01	0.42	0.73	0.08	0.003
*Enterococcus faecium*	0.64	ND	ND	ND	ND	0.08	0.001
*Weissella confusa*	0.53	ND	0.02	ND	ND	0.11	0.529

ND: not detected; CK: 2% glucose; T1: *Lactobacillus plantarum* at 3 × 10^8^ CFU/g + 2% glucose; T2: Glucanase at 833 U/g + *L. plantarum* at 3 × 10^8^ CFU/g + 2% glucose; T3: Cellulase at 533 U/g + *L. plantarum* at 3 × 10^8^ CFU/g + 2% glucose; T4: Glucanase at 833 U/g + cellulase at 533 U/g + *L. plantarum* at 3 × 10^8^ CFU/g + 2% glucose; SEM: standard error of the means

### Correlation between bacterial species and silage quality

**Fig. 3:**
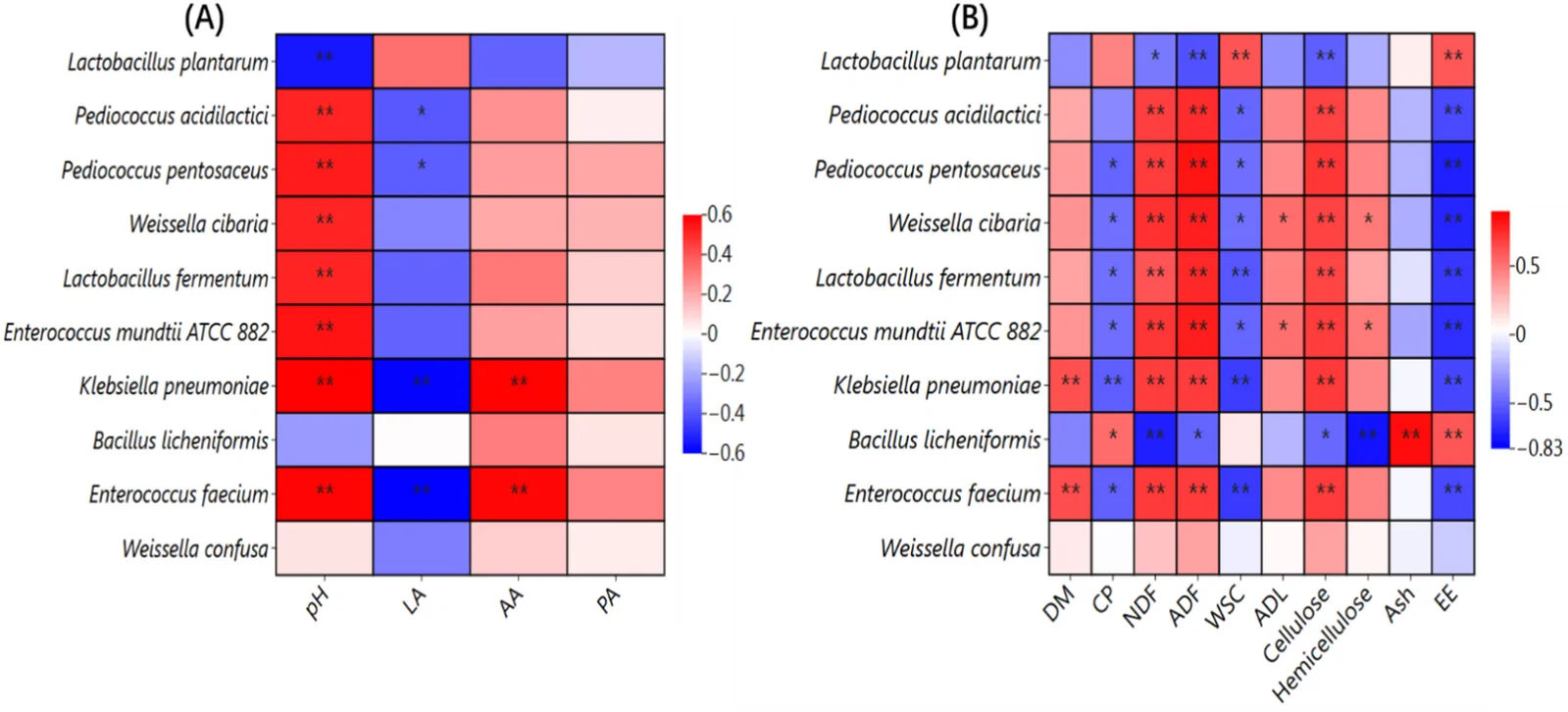
Heatmap of the Spearman correlation coefficients between dominant bacterial species (**A**) silage fermentation characteristics (**B**) nutrient composition parameters on day 42. Positive correlations are shown in red, and negative correlations are shown in blue. LA: lactic acid; AA: acetic acid; PA: propionic acid; DM: dry matter; CP: crude protein; NDF: neutral detergent fiber; ADF: acid detergent fiber; ADL: acid detergent lignin; WSC: water-soluble carbohydrates; EE: ether extract. * Significance at *P* < 0.05; ** significance at *P* < 0.01

Figure 3 illustrates the Spearman correlation analysis on Day 42 between dominant bacterial species and both silage fermentation characteristics and nutrient composition parameters. In Fig. 3A, most bacterial species exhibited highly positive correlations with pH (*P* < 0.01), and negative correlations with lactic acid. Conversely, *Lactobacillus plantarum* was the notable exception, showing a strong negative association with pH (*P* < 0.01) and a positive association with lactic acid. *Klebsiella pneumoniae* and *Enterococcus faecium* demonstrate negative correlations with lactic acid (*P* < 0.01) and strong positive correlations with acetic acid (*P* < 0.01). The nutrient composition heatmap (Fig. 3B) revealed clear relationships between bacterial species and silage nutritional characteristics. The most dominant bacteria, including *Pediococcus acidilactici*, *Pediococcus pentosaceus*, *Weissella cibaria*, *Lactobacillus fermentum*, and *Enterococcus mundtii ATCC 882*, were positively correlated with DM, NDF, ADF, ADL, cellulose, and hemicellulose, and were negatively correlated with CP, WSC, and EE. In contrast, *Lactobacillus plantarum* correlated positively with CP, WSC, and EE, while correlating negatively with NDF and ADF, ADL, cellulose, and hemicellulose. *Klebsiella pneumoniae* and *Enterococcus faecium* exhibited a positive relationship with fiber components and an inverse relationship with CP, WSC, and EE. *Bacillus licheniformis* displayed the opposite trend, correlating positively with CP and EE and negatively with most fiber fractions. *Weissella confusa* showed weak and non-significant correlations with both fermentation characteristics and nutrient composition.

## Discussion

### Effect of additives on the fermentation quality of cassava leaf silage

Silage pH is a key measure of fermentation efficiency and overall silage quality. Properly fermented silage generally exhibits a pH between 3.8 and 4.2 (Kung et al. 2018). Maintaining an acidic environment prevents the proliferation of spoilage organisms and ensures good fermentation quality (Keshri et al. 2018). In this study, the pH values decreased progressively as ensiling time increased, indicating active fermentation and rapid acidification of the silage mass. The enzyme-treated groups, particularly T3 and T4, exhibited the lowest pH values at Days 21 and 42, suggesting that cellulase and glucanase enhanced lactic acid fermentation by increasing the availability of fermentable substrates. This pH decline aligns with earlier reports indicating that cellulase enzymes and LAB stimulate the degradation of structural carbohydrates into fermentable sugars, thereby enhancing lactic acid production and improving silage preservation (Campana et al. 2022; Du et al. 2023). In line with the current results, Zhao et al. (2021) found that cellulase supplementation enhanced lactic acid production and lowered silage pH. Likewise, Li et al. (2023) found that adding cellulase and xylanase to corn silage significantly increased lactic acid concentration and reduced pH. The significant increase in lactic acid concentration in treated silages further confirmed enhanced fermentation quality. Treatments supplemented with additives produced higher lactic acid contents than the CK throughout the ensiling period, suggesting that enzyme-LAB combinations promoted efficient homofermentative activity. The greater lactic acid concentration observed in this study agrees with the results reported by Ma et al. (2023), who found that LAB and cellulase inoculation promote homofermentative fermentation, leading to greater lactic acid accumulation. The amounts of volatile fatty acids in silage can partly indicate nutrient losses and are commonly used to assess silage fermentation quality (Kung et al. 2018). Higher acetate in the silage may lead to lower energy value and reduced dry matter recovery (He et al. 2025). In the present study, the lower acetic acid in T1-T4 than in CK indicated that *L. plantarum* and enzyme inoculation successfully improved fermentation quality. In addition, propionic acid concentrations remained low across all treatments, which is considered favorable, as excessive propionic acid may indicate secondary fermentation or clostridial activity (Kung et al. 2018). The low propionic acid levels, together with reduced pH and elevated lactic acid concentrations, demonstrated that the silages underwent desirable fermentation.

### Effect of additives on the chemical composition and fiber components of cassava leaf silage

The changes in the chemical composition of cassava leaf silage during ensiling are closely linked to microbial fermentation activity and the combined effects of fiber-degrading enzymes and LAB inoculation. DM content gradually decreased with prolonged ensiling, particularly in enzyme- and inoculant-treated silages, indicating active microbial fermentation and utilization of soluble substrates. Similar reductions in DM during silage fermentation have been reported by Qiu et al. (2024), who noted that respiration and microbial metabolism contribute to DM losses. The increase in CP content in enzyme-treated silages compared with the control suggests that fibrolytic enzymes and microbial inoculants improved nitrogen preservation and reduced proteolysis. These results are consistent with those reported by Liu et al. (2025), who investigated the effects of *Lactobacillus plantarum*, cellulase, and xylanase supplementation on corn stover silage. Another study demonstrated that cellulase and LAB inoculation maintain higher CP content in oat silage (Xu et al. 2022). The higher CP content could be explained by fibrolytic enzymes, which break down structural carbohydrates into compounds that support the growth of LAB during fermentation. Increased lactic acid production by these bacteria lowers the pH, which reduces protein degradation by inhibiting both protein-degrading enzymes and undesirable microorganisms (Bai et al. 2021). The significant decreases in NDF, ADF, cellulose, and hemicellulose in treatments containing cellulase and glucanase indicate enhanced hydrolysis of structural carbohydrates. Fibrolytic enzymes degrade plant cell walls into fermentable sugars (Dong et al. 2025), thereby improving silage digestibility and fermentation quality (Kaewpila et al. 2021). Earlier research has established that the inclusion of cellulase during fermentation effectively lowers NDF and ADF concentrations (Su et al. 2019). According to another study, the addition of cellulase hydrolyzes lignocellulose, which substantially lowers the levels of NDF, ADF, hemicellulose, and cellulose in the silages (Xu et al. 2022). The lower fiber concentration may also increase nutrient digestibility and improve feed utilization by ruminants. In contrast, ADL content showed relatively smaller changes because lignin is resistant to microbial and enzymatic degradation during the ensiling process. This agrees with previous reports indicating that lignin is minimally affected by silage fermentation due to its complex phenolic structure (Qiu et al. 2024). Additionally, the increase in ash content may reflect concentration effects resulting from organic matter degradation during fermentation. WSC during ensiling is a critical indicator of fermentation efficiency and substrate availability for LAB. In this study, the progressive decline in WSC content over the ensiling period reflects the typical biological process in which fermentable sugars are consumed to produce organic acids, which lower the pH and stabilize the forage (Zahiroddini et al. 2004). Similar observations were reported by Xu et al. (2022) and Liu et al. (2025), who found that applying exogenous enzymes together with LAB inoculants promotes the breakdown of structural carbohydrates into readily fermentable sugars, thereby modifying WSC consumption during ensiling.

### Alpha and beta diversity of microorganisms in cassava leaf silage

In the current study, alpha diversity analysis indicated that the treatments had little effect on bacterial richness, as the ACE, Chao1, and observed species indices did not differ significantly among groups. In contrast, the CK treatment showed significantly greater Shannon and Simpson diversity indices than the treated groups, indicating lower bacterial diversity after additive application. This reduction in diversity suggests that the treatments promoted the dominance of beneficial bacteria, particularly lactic acid bacteria, during fermentation. Such a reduction in microbial diversity is generally considered beneficial during ensiling because rapid proliferation of desirable lactic acid bacteria restrains the proliferation of unwanted microbes and accelerates acidification. Our finding is consistent with previous studies reporting that additives such as microbial inoculants and exogenous enzymes reduce bacterial diversity while improving fermentation quality by creating a more selective acidic environment (Mu et al. 2023; Qi et al. 2026). Similar reductions in bacterial diversity have also been reported in alfalfa silages treated with lactic acid bacteria, where the dominance of *Lactobacillus* species was associated with improved preservation and fermentation efficiency (Yang et al. 2019).

The NMDS analysis of the cassava leaf silage bacterial community clearly demonstrates a distinct separation in beta diversity between the CK and the treatment groups (T1-T4). This observation aligns with previous findings, in which beta diversity analysis revealed clear separation among the treated groups in response to different additives (Li et al. 2020). This sharp divergence suggests that the treatments successfully altered the fermentation environment, potentially suppressing undesirable epiphytic bacteria and promoting beneficial strains. Close clustering of the treatment groups implies that the combined use of microbial inoculants and fibrolytic enzymes promotes a more consistent bacterial community during fermentation. Similar changes in bacterial community composition have been reported in mixed silage of king grass and cassava foliage, where additive-treated silages showed distinct microbial profiles compared with untreated controls and were dominated by beneficial lactic acid bacteria (Liu et al. 2022a). These findings demonstrate that the combined use of lactic acid bacteria and fibrolytic enzymes contributes to a more stable microbial ecosystem and improved silage fermentation.

### Relative abundance of microorganisms in cassava leaf silage

The phylum-level analysis of the cassava leaf silage bacterial community after 42 days of ensiling reveals a microbiome dominated by Bacillota, with relative abundances exceeding 92% across all treatment groups. This dominance is an indicator of a successful fermentation process, as Bacillota (formerly Firmicutes) is widely recognized as the primary phylum responsible for lactic acid fermentation in silage (Wang et al. 2026). This is consistent with findings in other forages, such as alfalfa, where Firmicutes constitute the majority of the bacterial community (Hu et al. 2020). Members of this phylum, particularly LAB, rapidly use WSC and produce lactic acid that lowers pH, inhibits unwanted microorganisms, and improves silage stability (Xu et al. 2024). In contrast, Pseudomonadota (synonym Proteobacteria) are commonly associated with fresh forage and early fermentation because many species require oxygen and are less tolerant of acidic, anaerobic conditions (Li et al. 2025). Therefore, the replacement of Pseudomonadota by Bacillota is considered an indicator of successful fermentation (Liu et al. 2024). Significant treatment effects were observed for Cyanobacteria, Actinomycetota, and Deinococcota. The marked reduction of Cyanobacteria in treated silages compared to the CK is a positive outcome, as Cyanobacteria are undesirable in silage and can be associated with poor fermentation quality (Ridwan et al. 2023). The decrease in Actinomycetota and Deinococcota in the treated groups may reflect the creation of a more selective environment that disfavors these phyla. Other phyla, such as Verrucomicrobiota, Bacteroidota, and Fusobacteria, were present in negligible amounts (≤ 0.51%), suggesting they are minor components of the silage ecosystem with limited functional roles in the fermentation process.

The predominance of *Lactobacillus plantarum* in the enzyme-treated silages indicates that the inoculated strain successfully became the dominant microorganism during fermentation. A high abundance of *L. plantarum* is widely associated with rapid acidification, efficient lactic acid production, and improved preservation of silage (Meng et al. 2025). This finding agrees with Liu et al. (2022b), who reported that *L. plantarum* dominated the bacterial community and enhanced fermentation quality. In contrast, the reduced abundance of *Pediococcus acidilactici* and *Pediococcus pentosaceus* suggests that these early colonizers were gradually replaced by *L. plantarum* as fermentation progressed. This microbial succession is common because *Pediococcus spp*. initiate acid production during the early stage but are less competitive under low-pH conditions (Ma et al. 2026). The lower abundance of heterofermentative species like *Weissella cibaria*, *Weissella confusa*, and *Lactobacillus fermentum* indicates a transition from heterofermentative to homofermentative fermentation, which favors greater lactic acid accumulation. However, they are usually suppressed as acidity increases because they cannot tolerate high-acid environments (Cai et al. 1998). Likewise, the decline of *Enterococcus mundtii* and *Enterococcus faecium* reflects the establishment of a more stable fermentation environment, as these bacteria are generally abundant during the initial stage but decrease as the silage environment becomes more acidic (Li et al. 2022). The absence of *Klebsiella pneumoniae* in the enzyme-treated silage is particularly beneficial because this undesirable microorganism may compete with LAB for available nutrients and can impair silage quality (Soundharrajan et al. 2025). Although *Bacillus licheniformis* remained at a low abundance, it may have contributed to fiber degradation through extracellular enzyme production without disrupting fermentation (Yu et al. 2025).

### Correlation between bacterial species and silage quality

The relationships linking dominant bacterial species to fermentation indices and nutrient composition indicate that the microbial community contributes substantially to shaping silage quality. In this study, *Lactobacillus plantarum* was strongly negatively associated with pH but positively associated with lactic acid concentration. This observation corresponds with the work of Mu et al. (2020), who showed that *L. plantarum* is a primary driver of rapid acidification in silage. The beneficial effects of *L. plantarum* are further supported by the findings of Li et al. (2026), who observed that this species promoted acidification, reduced nutrient losses, and enhanced silage stability. Conversely, several species including *Pediococcus acidilactici*, *Pediococcus pentosaceus*, *Weissella cibaria*, and *Enterococcus faecium* exhibited a positive relationship with pH but an inverse relationship with lactic acid concentration. Comparable associations have been documented by Hao et al. (2024) and Peng et al. (2024). Earlier studies generally describe these taxa as early-stage lactic acid producers that initiate silage fermentation; however, their activities often decline as pH drops (Sun et al. 2021). Regarding nutrient composition, *Pediococcus*, *Weissella*, and *Enterococcus* species displayed strong negative correlations with CP, WSC, and EE. These results agree with former studies reporting that epiphytic and inoculant bacteria continuously consume available sugars and soluble nutrients as energy sources during prolonged fermentation (Xu et al. 2022). The inverse relationship between certain bacterial species and fiber fractions aligns with research indicating that specific inoculants, like *Lactobacillus plantarum*, can enhance the preservation of CP while contributing to a reduction in fiber content (Jin et al. 2024).

## Conclusion

Co-inoculating cassava leaf silage with fiber-degrading enzymes and LAB markedly enhances its fermentation properties, nutritional parameters, and bacterial ecology. All additive treatments (T1–T4) yielded well-fermented silage exhibiting a lower pH and greater lactic acid content than the CK, indicating rapid acidification and successful preservation. Treated silages showed better nutrient conservation, with higher levels of CP and WSC. Additionally, enzyme treatments effectively enhanced fiber degradation, resulting in significant reductions in NDF, ADF, cellulose, and hemicellulose contents over the ensiling period. Microbial analysis revealed that additive treatments reduced bacterial diversity while promoting a more stable bacterial community dominated by *Lactobacillus plantarum*, which may have contributed to improved fermentation and the suppression of undesirable microorganisms. Among all treatments, T4, containing glucanase (833 U/g), cellulase (533 U/g), *L. plantarum* (3 × 10⁸ CFU/g), and 2% glucose, showed the best overall performance. Collectively, the synergistic combination of fibrolytic enzymes (cellulase and glucanase) and lactic acid bacteria (*Lactobacillus plantarum*) represents an effective strategy to optimize cassava leaf silage. This approach may increase the feeding value of cassava leaves and support their efficient use as high-quality ruminant feed.

## Supplementary Information

Below is the link to the electronic supplementary material.

Supplementary Material 1

## Data Availability

The datasets used and/or analyzed in the current study are available from the corresponding author on reasonable request.

## Abbreviations

LAB: Lactic acid bacteria
HCN: Hydrogen cyanide
DM: Dry matter
CP: Crude protein
EE: Ether extract
NDF: Neutral detergent fiber
ADF: Acid detergent fiber
ADL: Acid detergent lignin
WSC: Water-soluble carbohydrates
RFV: Relative feed value
DDM: Digestible dry matter
DMI: Dry matter intake
HPLC: High-performance liquid chromatography
CRD: Completely randomized design
ASVs: Amplicon sequence variants
QIIME2: Quantitative insights into microbial ecology 2
DADA2: Divisive amplicon denoising algorithm 2
NMDS: Non-metric multidimensional scaling
GLM: General linear model
SPSS: Statistical package for the social sciences
SEM: Standard error of the mean
CK: Control group

## References

[CR1] AOAC (2000) Official methods of analysis (17th ed). Association of official analytical chemists, Gaithersburg, Maryland, United States of America

[CR3] Bai J, Ding Z, Ke W, Xu D, Wang M, Huang W, Zhang Y, Liu F, Guo X (2021) Different lactic acid bacteria and their combinations regulated the fermentation process of ensiled alfalfa: ensiling characteristics, dynamics of bacterial community and their functional shifts. Microb Biotechnol 14(3):1171–1182. 10.1111/1751-7915.13785 PMC8085944 33666350

[CR2] Bai B, Qiu R, Sun L, Bao J, Liu Y, Ge G, Jia Y, Wang Z (2024) Effect isolated lactic acid bacteria inoculation on the quality, bacterial composition and metabolic characterization of Caragana korshinskii silage. Chem Biol Technol Agric 11(1):67. 10.1186/s40538-024-00591-z

[CR4] Bakare AG, Zindove TJ, Bhavna A, Devi A, Takayawa SL, Sharma AC, Iji PA (2023) Lactobacillus buchneri and molasses can alter the physicochemical properties of cassava leaf silage. Heliyon 9(11):e22141. 10.1016/j.heliyon.2023.e22141 PMC10685371 38034723

[CR5] Burns A, Gleadow R, Cliff J, Zacarias A, Cavagnaro T (2010) Cassava: the drought, war and famine crop in a changing world. Sustainability 2(11):3572–3607. 10.3390/su2113572

[CR6] Cai Y, Benno Y, Ogawa M, Ohmomo S, Kumai S, Nakase T (1998) Influence of Lactobacillus spp. from an Inoculant and of Weissella and Leuconostoc spp. from Forage Crops on Silage Fermentation. Appl Environ Microbiol 64(8):2982–2987. 10.1128/AEM.64.8.2982-2987.1998 PMC106803 9687461

[CR7] Callahan BJ, McMurdie PJ, Rosen MJ, Han AW, Johnson AJA, Holmes SP (2016) DADA2: High-resolution sample inference from Illumina amplicon data. Nat Methods 13(7):581–583. 10.1038/nmeth.3869 PMC4927377 27214047

[CR8] Campana M, Morais J, Capucho E, Garcia T, Pedrini C, Gandra J, Del Valle T (2022) Fibrolytic enzymes increase fermentation losses and reduce fiber content of sorghum silage. Ann Anim Sci. 10.2478/aoas-2022-0038

[CR9] Chen L, Li J, Dong Z, Shao T (2019) Effects of lactic acid bacteria inoculants and fibrolytic enzymes on the fermentation quality, in vitro degradability, ruminal variables and microbial communities of high-moisture alfalfa silage. Grassl Sci 65(4):216–225. 10.1111/grs.12240

[CR10] Dagaew G, Cheas S, Suntara C, Supapong C, Cherdthong A (2025) Effects of Replacing soybean meal with enzymatically fermented citric waste pellets on in vitro rumen fermentation, degradability, and gas production kinetics. Animals 15(16):2351. 10.3390/ani15162351 PMC12382709 40867679

[CR11] Dong D, Zhang L, Zhao J, Dong Z, Li J, Shao T (2025) Synergistic effects of exogenous lactobacillus plantarum and fibrolytic enzymes on fermentation quality, fiber degradation, and in vitro digestibility of napiergrass (*Pennisetum purpureum*) silage. Agronomy 15(2):340. 10.3390/agronomy15020340

[CR12] Du Z, Yamasaki S, Oya T, Cai Y (2023) Cellulase–lactic acid bacteria synergy action regulates silage fermentation of woody plant. Biotechnol Biofuels Bioprod 16(1):125. 10.1186/s13068-023-02368-2 PMC10403842 37542284

[CR13] Edgar RC (2010) Search and clustering orders of magnitude faster than BLAST. Bioinformatics 26(19):2460–2461. 10.1093/bioinformatics/btq461 20709691

[CR14] Goering HK, Van Soest PJ (1970) Forage fiber analyses (apparatus, reagents, procedures, and some applications). Agric. Handbook No. 379. agricultural research service-USDA, Washington, DC

[CR15] Hao L, Jiang F, Wang Y, Wang H, Hu H, You W, Hu X, Cheng H, Wang C, Song E (2024) Formic acid enhances whole-plant mulberry silage fermentation by boosting lactic acid production and inhibiting harmful bacteria. Front Microbiol. 10.3389/fmicb.2024.1399907 PMC11194324 38915298

[CR16] He L, Jiang C, Dong H, Wang Y, Tang J, Hu M, Luo J, Du S, Jia Y, Xiao Y, You S (2025) Effects of cellulase or Lactobacillus plantarum on ensiling performance and bacterial community of sorghum straw. BMC Microbiol 25(1):300. 10.1186/s12866-025-03982-w PMC12083020 40380109

[CR17] Hossain MD, Yan Q, Zhou Z, Zhang X, Wittayakun S, Napasirth V, Napasirth P, Lukuyu BA, Tan Z (2025) Cassava as a feedstuff for ruminant feeding system in Belt and Road countries: innovations, benefits and challenges. Agric Food Res 21:101874. 10.1016/j.jafr.2025.101874

[CR18] Hu Z, Niu H, Tong Q, Chang J, Yu J, Li S, Zhang S, Ma D (2020) The microbiota dynamics of alfalfa silage during ensiling and after air exposure, and the metabolomics after air exposure are affected by lactobacillus casei and cellulase addition. Front Microbiol. 10.3389/fmicb.2020.519121 PMC7732661 33329411

[CR19] Jin S, Tahir M, Huang F, Wang T, Li H, Shi W, Liu Y, Liu W, Zhong J (2024) Fermentation quality, amino acids profile, and microbial communities of whole-plant soybean silage in response to Lactiplantibacillus plantarum B90 alone or in combination with functional microbes. Front Microbiol.. 10.3389/fmicb.2024.1458287 PMC11631902 39664048

[CR20] Kaewpila C, Gunun P, Kesorn P, Subepang S, Thip-uten S, Cai Y, Pholsen S, Cherdthong A, Khota W (2021) Improving ensiling characteristics by adding lactic acid bacteria modifies in vitro digestibility and methane production of forage-sorghum mixture silage. Sci Rep 11(1):1968. 10.1038/s41598-021-81505-z PMC7820244 33479407

[CR21] Keshri J, Chen Y, Pinto R, Kroupitski Y, Weinberg ZG, Sela S (2018) Microbiome dynamics during ensiling of corn with and without Lactobacillus plantarum inoculant. Appl Microbiol Biotechnol 102(9):4025–4037. 10.1007/s00253-018-8903-y 29536147

[CR23] Kim M, Chun J (2014) Chap. 4–16S rRNA Gene-based identification of bacteria and archaea using the EzTaxon server. In Goodfellow M, Sutcliffe I, Chun J (Eds.), Method Microbiol 41. Academic Press. 61–74 10.1016/bs.mim.2014.08.001

[CR22] Kim D, Lee KD, Choi KC (2021) Role of LAB in silage fermentation: effect on nutritional quality and organic acid production—an overview. AIMS Agric Food 6(1):216–234. 10.3934/agrfood.2021014

[CR24] Kung L, Shaver RD, Grant RJ, Schmidt RJ (2018) Silage review: interpretation of chemical, microbial, and organoleptic components of silages. J Dairy Sci 101(5):4020–4033. 10.3168/jds.2017-13909 29685275

[CR25] Leyva A, Quintana A, Sánchez M, Rodríguez EN, Cremata J, Sánchez JC (2008) Rapid and sensitive anthrone–sulfuric acid assay in microplate format to quantify carbohydrate in biopharmaceutical products: Method development and validation. Biologicals 36(2):134–141. 10.1016/j.biologicals.2007.09.001 18042399

[CR31] Li M, Zi X, Zhou H, Lv R, Tang J, Cai Y (2019) Silage fermentation and ruminal degradation of cassava foliage prepared with microbial additive. AMB Express 9(1):180. 10.1186/s13568-019-0906-2 PMC6842376 31707488

[CR29] Li M, Zhang L, Zhang Q, Zi X, Lv R, Tang J, Zhou H (2020) Impacts of citric acid and malic acid on fermentation quality and bacterial community of cassava foliage silage. Front Microbiol 10.3389/fmicb.2020.595622 PMC7793898 33424799

[CR30] Li M, Zi X, Zhou H, Lv R, Tang J (2021) Effect of lactic acid bacteria, molasses, and their combination on the fermentation quality and bacterial community of cassava foliage silage. Anim Sci J. 10.1111/asj.13635 34626133

[CR28] Li M, Yu Q, Xu J, Sun H, Cheng Q, Xie Y, Wang C, Li P, Chen C, Zheng Y (2022) Effect of different organic acid additives on the fermentation quality and bacterial community of paper mulberry (*Broussonetia papyrifera*) silage. Front Microbiol. 10.3389/fmicb.2022.1038549 PMC9665874 36386675

[CR32] Li S, Wang H, Luo M, Wu B, Duan H, Ma X, Ren J (2023) Effects of cellulase and xylanase additives on fermentation quality and nutrient composition of silage maize. Circular Agric Syst. 10.48130/CAS-2023-0008

[CR26] Li C, Nair J, Chevaux E, McAllister TA, Wang Y (2025) Effect of inoculation of lactic acid bacteria and fibrolytic enzymes on microbiota in the terminal and aerobically exposed short-growing season whole-plant corn silage. Fermentation 11(9):530. 10.3390/fermentation11090530

[CR27] Li E, Na N, Wu N, Ke W, Bai C, Xue Y (2026) Dynamics of bacterial community and fermentation quality of wheat straw silage treated with lactic acid bacteria and/or sucrose. Anim Feed Sci Technol 331:116598. 10.1016/j.anifeedsci.2025.116598

[CR37] Liu Y, Chen T, Sun R, Zi X, Li M (2022a) The effects of lactic acid bacteria and molasses on microbial community and fermentation performance of mixed silage of king grass and cassava foliage. Front Anim Sci. 10.3389/fanim.2022.879930

[CR38] Liu Y, Chen T, Sun R, Zi X, Li M (2022b) Effects of lactobacillus plantarum on silage fermentation and bacterial community of three tropical forages. Front Anim Sci. 10.3389/fanim.2022.878909

[CR36] Liu W, Si Q, Sun L, Wang Z, Mingjian L, Du S, Ge G, Jia Y (2023) Effects of cellulase and xylanase addition on fermentation quality, aerobic stability, and bacteria composition of low water-soluble carbohydrates oat silage. Fermentation 9:638. 10.3390/fermentation9070638

[CR35] Liu J, Zhao M, Hao J, Yan X, Fu Z, Zhu N, Jia Y, Wang Z, Ge G (2024) Effects of temperature and lactic acid Bacteria additives on the quality and microbial community of wilted alfalfa silage. BMC Plant Biol 24(1):844. 10.1186/s12870-024-05501-x PMC11382506 39251915

[CR34] Liu J, Liu M, Sheng P, Song C, Ma W, Bai B, Zhao J, Du S, Ge G, Wang Z, Jia Y (2025) Biotechnological effects of lactobacillus plantarum, cellulase, and xylanase on nutritional quality and microbial community structure of corn stover silage. Fermentation 11(1):14. 10.3390/fermentation11010014

[CR33] Liu H, Ouyang J, Zhao J, Yuan X, Shao T, Melak S, Li J (2026) Harnessing enzymatic-bacterial synergy for hybrid Pennisetum: an integrated meta-analysis and experimental validation from ensiling to enhanced methane production. Ind Crops Prod 239:122456. 10.1016/j.indcrop.2025.122456

[CR39] Ma J, Fan X, Wu T, Zhou J, Huang H, Qiu T, Xing Z, Zhao Z, Yin F, Gan S (2023) Lactic acid bacteria and cellulase improve the fermentation characteristics, aerobic stability and rumen degradation of mixed silage prepared with amaranth and rice straw. Fermentation 9:853. 10.3390/fermentation9090853

[CR40] Ma W, Bai B, Liu M, Sheng P, Bao J, Song C, Liu J, Ma H, Du S, Ge G, Jia Y, Wang Z (2026) Synergistic effects of cellulase and lactic acid bacteria(Pediococcus pentosaceus and Levilactobacillus brevis) on alfalfa silage fermentation and microbial dynamics. BMC Microbiol 26(1):417. 10.1186/s12866-026-04725-1 PMC13126710 41864867

[CR42] Meng H, Xu Y, Wang L, Wang J, Wang B, Wu H, Hou D, Wang S, Tong X, Jiang Y, Wang S (2025) Impact of Lactiplantibacillus plantarum on the fermentation quality, nutritional enhancement, and microbial dynamics of whole plant soybean silage. Front Microbiol 16:1565951. 10.3389/fmicb.2025.1565951 PMC12127759 40458705

[CR44] Mu L, Xie Z, Hu L, Chen G, Zhang Z (2020) Cellulase interacts with Lactobacillus plantarum to affect chemical composition, bacterial communities, and aerobic stability in mixed silage of high-moisture amaranth and rice straw. Bioresour Technol 315:123772. 10.1016/j.biortech.2020.123772 32653750

[CR43] Mu L, Wang Q, Wang Y, Zhang Z (2023) Effects of cellulase and xylanase on fermentative profile, bacterial diversity, and in vitro degradation of mixed silage of agro-residue and alfalfa. Chem Biol Technol Agric 10(1):40. 10.1186/s40538-023-00409-4

[CR45] Napasirth V, Napasirth P, Sulinthone T, Phommachanh K (2015) Microbial population, chemical composition and silage fermentation of cassava residues. Anim Sci J. 10.1111/asj.12362 25781881

[CR46] Ndubuisi N, Ano C (2018) Cyanide in cassava: a review. Int J Genom Data Min 2(1):118. 10.29011/2577-0616.000118

[CR47] Peng S, Xie L, Cheng Y, Wang Q, Feng L, Li Y, Lei Y, Sun Y (2024) Effect of Lactiplantibacillus and sea buckthorn pomace on the fermentation quality and microbial community of paper mulberry silage. Front Plant Sci. 10.3389/fpls.2024.1412759 PMC11395498 39280944

[CR48] Qi M, Wang Z, Jia Y, Ge G (2026) Effects of additives on fermentation quality, nutritional quality, and microbial diversity of Leymus chinensis Silage. Microorganisms 14(1):27. 10.3390/microorganisms14010027 PMC12843975 41597547

[CR49] Qiu C, Liu N, Diao X, He L, Zhou H, Zhang W (2024) Effects of cellulase and xylanase on fermentation characteristics, chemical composition and bacterial community of the mixed silage of king grass and rice Straw. Microorganisms 12(3):561. 10.3390/microorganisms12030561 PMC10972424 38543612

[CR50] Quast C, Pruesse E, Yilmaz P, Gerken J, Schweer T, Yarza P, Peplies J, Glöckner FO (2013) The SILVA ribosomal RNA gene database project: improved data processing and web-based tools. Nucleic Acids Res 41(D1):D590–D596. 10.1093/nar/gks1219 PMC3531112 23193283

[CR51] Rashid M, Aboshady HM, Soltan YA, Archimede H, Ghoneem WMA (2025) Phytochemical profile and in vitro evaluation of cassava (Manihot esculenta Crantz) foliage as ruminant feed with/without green banana flour. Sci Rep 15(1):721. 10.1038/s41598-024-82450-3 PMC11699222 39753594

[CR52] Ridwan R, Abdelbagi M, Sofyan A, Fidriyanto R, Astuti WD, Fitri A, Sholikin MM, Rohmatussolihat, Sarwono KA, Jayanegara A, Widyastuti Y (2023) A meta-analysis to observe silage microbiome differentiated by the use of inoculant and type of raw material. Front Microbiol 14:1063333. 10.3389/fmicb.2023.1063333 PMC9998704 36910222

[CR53] Schloss PD, Westcott SL, Ryabin T, Hall JR, Hartmann M, Hollister EB, Lesniewski RA, Oakley BB, Parks DH, Robinson CJ, Sahl JW, Stres B, Thallinger GG, Horn DJV, Weber CF (2009) Introducing mothur: open-source, platform-independent, community-supported software for describing and comparing microbial communities. Appl Environ Microbiol 75(23):7537–7541. 10.1128/AEM.01541-09 PMC2786419 19801464

[CR54] Soundharrajan I, Min C-W, Jung JS, Choi KC (2025) Effects of the novel Lacticaseibacillus paracasei K-68 inoculant on nutrient content, fermentation, and microbial dynamics changes in Dacheongok Corn Silage. Fermentation 11(6):304. 10.3390/fermentation

[CR55] Su R, Ni K, Wang T, Xiaopan Y, Zhang J, Liu Y, Shi W, Yan L, Chen J, Zhong J (2019) Effects of ferulic acid esterase-producing Lactobacillus fermentum and cellulase additives on the fermentation quality and microbial community of alfalfa silage. PeerJ 7:e7712. 10.7717/peerj.7712 PMC6788448 31608168

[CR56] Sun L, Bai C, Xu H, Na N, Jiang Y, Yin G, Liu S, Xue Y (2021) Succession of bacterial community during the initial aerobic, intense fermentation, and stable phases of whole-plant corn silages treated with lactic acid bacteria suspensions prepared From Other Silages. Front Microbiol. 10.3389/fmicb.2021.655095 PMC8032959 33841382

[CR41] Van Hans MN W (2001) Cassava tops ensiled with or without molasses as additive effects on quality, feed intake and digestibility by heifers. Asian-Australas J Anim Sci 14(5):624–630. 10.5713/ajas.2001.624

[CR57] Van Soest PJ, Robertson JB, Lewis BA (1991) Methods for dietary fiber, neutral detergent fiber, and nonstarch polysaccharides in relation to animal nutrition. J Dairy Sci 74(10):3583–3597. 10.3168/jds.S0022-0302(91)78551-2 1660498

[CR58] Vuma M, Ratsaka MM, Tjelele JT, Langa T, Nkosi BD, Malebana IMM (2026) Effects of protein supplementation and microbial inoculation on fermentation quality, aerobic stability, and in vitro digestibility of maize silage. Fermentation 12(3):155. 10.3390/fermentation12030155

[CR60] Wang S, Guo G, Li J, Chen L, Dong Z, Shao T (2019) Improvement of fermentation profile and structural carbohydrate compositions in mixed silages ensiled with fibrolytic enzymes, molasses and Lactobacillus plantarum MTD-1. Ital J Anim Sci 18(1):328–335. 10.1080/1828051X.2018.1528899

[CR59] Wang Q, Wang R, Wang C, Dong W, Zhang Z, Zhao L, Zhang X (2022) Effects of cellulase and lactobacillus plantarum on fermentation quality, chemical composition, and microbial community of mixed silage of whole-plant corn and peanut vines. Appl Biochem Biotechnol 194(6):2465–2480. 10.1007/s12010-022-03821-y 35132520

[CR61] Wang Y, Huang X, Liao C, Tang X, Hong T, Zhang Y, Wang P, Chen C, Li P (2026) Effects of lactic acid bacteria from pickles on the silage fermentation and bacterial community and anerobic stability of maize, soybean and their mixture in karst regions. Microorganisms 14(3):528. 10.3390/microorganisms14030528 PMC13029001 41900287

[CR62] Wu Qiufei XH, Wang Qinfei LL, Li Kaimian ZZ (2017) A comprehensive review of the utilization of cassava (manihot esculenta crantz) leaf. Agric Sci Technol 18(12):2576–2586

[CR64] Xu J, Zhang K, Lin Y, Li M, Wang X, Yu Q, Sun H, Cheng Q, Xie Y, Wang C, Li P, Chen C, Yang F, Zheng Y (2022) Effect of cellulase and lactic acid bacteria on the fermentation quality, carbohydrate conversion, and microbial community of ensiling oat with different moisture contents. Front Microbiol. 10.3389/fmicb.2022.1013258 PMC9581316 36274697

[CR63] Xu J, Ma J, Sa R, Sui H, Wang X, Li Q, Zhu X, Wu B, Hu Z, Niu H (2024) Effects of lactic acid bacteria inoculants on the nutrient composition, fermentation quality, and microbial diversity of whole-plant soybean-corn mixed silage. Front Microbiol 15:1347293. 10.3389/fmicb.2024.1347293 PMC11056550 38686105

[CR65] Yang L, Yuan X, Li J, Dong Z, Shao T (2019) Dynamics of microbial community and fermentation quality during ensiling of sterile and nonsterile alfalfa with or without Lactobacillus plantarum inoculant. Bioresour Technol 275:280–287. 10.1016/j.biortech.2018.12.067 30594838

[CR66] Yu D, Lee J-E, Rhee M-S, Shim S-M, Om A-S, Yu H, Kook M (2025) Isolation of biofilm-forming bacteria from food processing equipment surfaces and the biofilm-degrading activity of bacillus licheniformis YJE5. Foods 14(21):3592. 10.3390/foods14213592 PMC12607818 41227566

[CR67] Zahiroddini H, Baah J, Absalom W, McAllister T (2004) Effect of an inoculant and hydrolytic enzymes on fermentation and nutritive value of whole crop barley silage. Anim Feed Sci Technol 117:317–330. 10.1016/j.anifeedsci.2004.08.013

[CR68] Zhao C, Wang L, Ma G, Jiang X, Yang J, Lv J, Zhang Y (2021) Cellulase interacts with lactic acid bacteria to affect fermentation quality, microbial community, and ruminal degradability in mixed silage of soybean residue and corn stover. Animals 11(2):334. 10.3390/ani11020334 PMC7912217 33525728

